# Dynamic alterations of circulating lymphocytes during the trajectory of Hantaan virus-induced hemorrhagic fever with renal syndrome

**DOI:** 10.3389/fimmu.2025.1567306

**Published:** 2025-05-29

**Authors:** Lin Su, Shuangjuan Liu, Lei Shi, Yuan Cheng, Juanjuan Gao, Ruirui Guo, Yinli He, Linpei Zhang, Tianyan Chen, Jinsong Hu, Xiaojiao Li, Yawen Wang

**Affiliations:** ^1^ Infectious Diseases Department, The First Affiliated Hospital of Xi’an Jiaotong University, Xi’an, Shaanxi, China; ^2^ BioBank, The First Affiliated Hospital of Xi’an Jiaotong University, Xi’an, Shaanxi, China; ^3^ Shaanxi Engineering Research Center for Biobank and Advanced Medical Research, The First Affiliated Hospital of Xi’an Jiaotong University, Xi’an, Shaanxi, China; ^4^ Medical Laboratory, Weinan Central Hospital, Weinan, Shaanxi, China; ^5^ Department of Breast Surgery, The First Affiliated Hospital of Xi’an Jiaotong University, Xi’an, Shaanxi, China; ^6^ Department of Cell Biology and Genetics, School of Basic Medical Sciences, Xi’an Jiaotong University, Xi’an, Shaanxi, China; ^7^ Department of Laboratory Medicine, First Affiliated Hospital, School of medicine, Xi’an Jiaotong University, Xi’an, Shaanxi, China

**Keywords:** dynamic alterations, lymphocyte subsets, biomarkers, HTNV, HFRS

## Abstract

**Introduction:**

Hemorrhagic fever with renal syndrome (HFRS) is a zoonotic disease with high mortality. Almost 90% of global cases of HFRS are induced by Hantaan virus (HTNV) infection. Although lymphocyte dysfunction is a critical factor in HFRS progression, the specific immune dynamics of HTNV remain unexplored, and current analyses predominantly depend on single-time point sampling. Therefore, comprehensive longitudinal studies are needed to characterize circulating lymphocyte dynamics during HTNV-induced HFRS progression.

**Methods:**

In this study, we conducted a flow cytometric analysis of circulating lymphocytes in 39 patients with HTNV-induced HFRS across different clinical phases. The analysis encompassed conventional T cells, unconventional T cells, B cells, NK cells and their respective repertoires.

**Results and Discussion:**

Here, we revealed phase-specific immune patterns: CD8^+^ T, CD8^+^ Tems, and activated CD8^+^ T, MAIT and NKT cells peaked during febrile/oliguric phases before declining in polyuria/recovery, while CD4^+^ T and MAIT cells showed inverse fluctuation patterns. Higher frequencies of CD8^+^ Tem, B, and CD56^dim^ NK cells during the febrile phase correlated with severe disease, enabling early risk stratification. Lower CD4^+^ Tcm levels in the oliguric phase marked progression to severe HFRS, indicating potential therapeutic strategies aimed at enhancing CD4^+^ Tcm generation or inhibiting effector differentiation. Additionally, CD38 and CD161 expression predicted specific lymphocyte subset dynamics, offering novel biomarkers for immunomodulatory strategies. Our study thus provides the first comprehensive atlas of lymphocyte evolution in HTNV-induced HFRS, connecting immune dysregulation with clinical outcomes.

## 1 Introduction

Hemorrhagic fever with renal syndrome (HFRS) is a zoonotic illness caused by Hantavirus, a type of negative-sense, single-stranded, enveloped RNA virus (1). Over 150,000 cases of HFRS are reported in more than 70 countries every year (2), with a case fatality rate ranging from 5%-15% (3). In Europe, the primary etiological agent of HFRS is the Puumala hantavirus (PUUV) (2, 4), whereas in China, which accounts for almost 90% of global cases, the disease is predominantly caused by the Hantaan virus (HTNV) (5, 6). Fever, thrombocytopenia and acute renal dysfunction are the main clinical characteristics of HFRS, and several sequential stages including fever, oliguria, polyuria, and recovery are always involved in the trajectory of HFRS (2, 7). However, due to a lack of understanding of the pathological mechanisms, current therapies for HFRS are limited to symptomatic treatments and supportive care (8).

Lymphocyte dysfunction is believed to be a critical factor in the progression of HFRS (9). T lymphocyte response has been observed during the acute phase of HFRS (10–12). Additionally, rapid and efficacious humoral responses have been documented in HFRS patients, with IgG levels being correlated with disease severity (13, 14). However, two significant gaps still exist in the current body of research. Firstly, the immunological profile of HTNV-induced HFRS, which accounts for the vast majority of global cases, is considerably understudied in comparison to PUUV-associated HFRS. This discrepancy limits our understanding of disease mechanisms in regions with high prevalence, such as China. Secondly, existing studies on lymphocytes in HFRS predominantly utilize single-timepoint sampling, which inadequately captures the evolution of immune responses across the distinct clinical phases of the disease.

To address these gaps, we performed longitudinal profiling of circulating lymphocytes in 39 HTNV-induced HFRS patients across different clinical phases, aiming to (1): Characterize the phase-dependent evolution of lymphocyte subsets, including conventional and unconventional T cells, B cells, NK cells, and their associations with disease severity; and (2) Identify predictive surface markers for lymphocyte fluctuations, thereby offering mechanistic insights and potential therapeutic targets. The results of the study are presented here.

## 2 Materials and methods

### 2.1 Study participants

This study recruited 39 patients diagnosed with HFRS at the Weinan Central Hospital, while 17 healthy donors were recruited as the control group at the First Affiliated Hospital of Xi’an Jiaotong University. The study was approved by the ethics committees of both the Weinan Central Hospital and the First Affiliated Hospital of Xi’an Jiaotong University. Written informed consent was obtained from all participants prior to the initial blood sample collection.

Patients with HFRS were diagnosed based on their clinical manifestation, epidemiological data and laboratory examination according to the criteria outlined in the Prevention and Treatment Strategy of HFRS published by the Ministry of Health, PR China. The clinical course of HFRS typically progresses through sequential phases: fever, oliguria, polyuria, and recovery. Comprehensive sampling was conducted at various stages, resulting in the successful collection of 39 blood samples during fever, 17 during oliguria, 25 during polyuria, and 14 during the recovery phase. The patients were categorized into two subgroups based on the severity of HFRS: moderate (n=26) and severe (n=13). Moderate HFRS is characterized by a body temperature ≤40°C accompanied by mucocutaneous hemorrhages (petechiae/ecchymoses) or conjunctival edema; urine protein levels ranging from + to +++; oliguria lasting ≤5 days or absence of oliguria; absence of persistent hypotension or presence of transient hypotension (systolic pressure 70–90 mmHg with pulse pressure <30 mmHg). Severe HFRS is defined by a body temperature >40°C with neurological symptoms; shock; oliguria lasting ≥5 days or anuria. 7 out of the 13 severe cases (53.85%) required hemodialysis due to refractory renal failure. Healthy individuals with no detectable abnormalities in comprehensive clinical and laboratory assessments were included as the control group. The age distributions of the subgroups were as follows: moderate cases (44.00 ± 17.06 years), severe cases (46.15 ± 18.20 years), and healthy individuals (47.47 ± 16.02 years). No significant differences in demographic variables of age were observed among the subgroups.

### 2.2 Sample collection

Peripheral blood samples were obtained from healthy donors and HFRS patients using vacuum-based blood collectors containing ethylenediaminetetraacetic acid (EDTA). In HFRS patients, blood samples were collected promptly upon entry into each clinical phase (fever, oliguria, polyuria, and recovery). The blood samples were processed within 6 hours of collection.

### 2.3 Flow cytometry analysis

Flow cytometry analysis was used to monitor the dynamic changes of lymphocyte subsets. Briefly, 50 μL of whole blood was incubated with a panel of directly conjugated monoclonal antibodies or unstained controls for 30 min at room temperature. Then, 450 μL lysing solution (Tiangen, China) was added to lyse the remaining erythrocytes for 10 min. The stained cells were then washed twice with ice-cold phosphate-buffered saline (PBS) and centrifuged at 1500 rpm for 5 min at 4°C. After that, the stained cells were resuspended in 400 μL PBS and immediately examined using a flow cytometer equipped with FACSDiva software (BD FACS celesta, BD Biosciences, USA). Data analysis was conducted using FlowJo software (version 10.8.1). The cell size gate was first established based on both forward scatter and side scatter to selectively include the lymphocyte population while excluding cellular debris and clumps.

### 2.4 Antibodies for flow cytometry analysis

Six different combinations of commercially fluorochrome-labeled antibodies against the following cell surface structures were used: (1) APC/Cyanine7 anti-human CD3, APC anti-human CD4, PerCP/Cyanine5.5 anti-human CD127 (lL-7Ra), PE anti-human CD25, Alexa Fluor^®^ 488 anti-human CD45RA, PE/Cyanine7 anti-human CD197 (CCR7); (2) APC/Cyanine7 anti-human CD3, PerCP/Cyanine5.5 anti-human CD8, Alexa Fluor^®^ 488 anti-human CD45RA, PE/Cyanine7 anti-human CD197 (CCR7); (3) APC/Cyanine7 anti-human CD3, PerCP/Cyanine5.5 anti-human CD8, PE/Cyanine7 anti-human CD56 (NCAM), APC anti-human CD161, FITC anti-human CD38, PE anti-human CD279 (PD-1); (4) APC/Cyanine7 anti-human CD3, PE anti-human CD19, FITC anti-human CD38, APC anti-human CD27; (5) APC/Cyanine7 anti-human CD3, PE/Cyanine7 anti-human CD56 (NCAM), Alexa Fluor^®^ 488 anti-human CD16, APC anti-human CD159a (NKG2A), PE anti-human CD159c (NKG2C); (6) APC/Cyanine7 anti-human CD3, FITC anti-human TCR γ/δ, PE/Cyanine7 anti-human TCR Vδ1, PerCP/Cyanine5.5 anti-human TCR Vδ2, PE anti-human CD27, APC anti-human CD45RA. The IgG isotype-matched antibody was applied as negative control to confirm the antibody specificity. All these antibodies were obtained from Biolegend (CA, USA), except for TCR Vδ1-PE-Cy7 from eBioscience (CA, USA). The gating strategy for definition of different lymphocyte subsets was shown in **Supplementary Figure S1**.

### 2.5 Statistical analysis

Statistics values are presented as means ± SD. All the statistical analyses were performed using GraphPad Prism (version 9; San Diego, CA, USA). For comparisons among multiple independent variables, either a one-way analysis of variance (ANOVA) or a Kruskal–Wallis test with Dunn’s multiple comparison was used, contingent upon the fulfillment of the normality assumption and homogeneity of variance. For comparisons specifically between two groups, the Mann-Whitney U test was applied. Relationships were assessed using the Spearman correlation coefficient. Linear mixed-effects model analysis was employed to analyze longitudinal trajectories of lymphocyte subsets between severe and moderate HFRS cohorts. *P* < 0.05 is considered statistically significant.

## 3 Results

### 3.1 Dynamic alterations of conventional T cells during the trajectory of HTNV-induced HFRS

**Figure 1:**
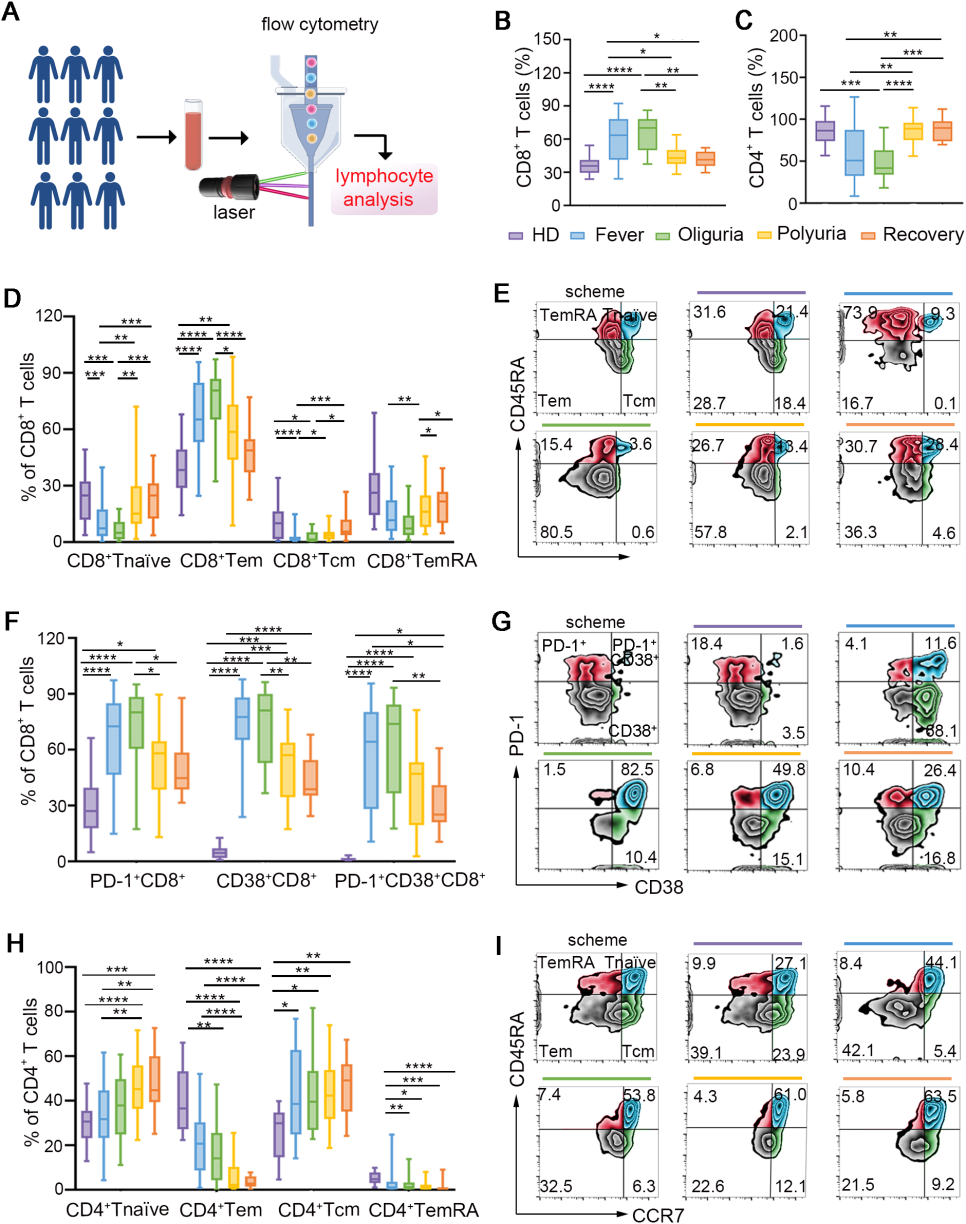
Dynamic perturbations of conventional T cells during the progression of HTNV-induced HFRS. **(A)** Scheme for circulating lymphocyte analysis. **(B, C)** The frequencies of CD8^+^ T cells **(B)** and CD4^+^ T cells **(C)** in the context of HTNV-induced HFRS. **(D)** Fluctuations of CD8^+^ T cell repertoires. **(E)** Representative FACS analysis of the phenotype of CD8^+^ T cell subsets (gated on CD3^+^CD8^+^ cells). **(F, G)** Subsets of CD4^+^ T cells following HTNV infection. **(H, I)** The activation of CD8^+^ T cells in each group (gated on CD3^+^CD8^+^ cells). Data are represented as mean ± SD. One-way ANOVA or the Kruskal–Wallis test with Dunn’s multiple comparison *post-hoc* test. *p< 0.05, **p< 0.01, ***p< 0.001, ****p< 0.0001.

CD8^+^ and CD4^+^ T cells play essential roles in antiviral immune responses (15). Our data revealed a significant increase in the proportion of CD8^+^ T cells during the fever and oliguria phases (**Figures 1A, B**), indicating that the expansion of CD8^+^ T cells was triggered by the presentation of neoantigens by HTNV. However, during the polyuria and recovery stages, CD8^+^ T cell frequencies reduced to levels comparable to those of healthy donors (**Figure 1B**), suggesting successful eradication of the virus in the late stages of HFRS. In contrast, a significant reduction in CD4^+^ T cells was noted during the fever and oliguria phases, with a subsequent rise during the polyuria and recovery phases (**Figure 1C**). This fluctuation may be linked to the destruction on CD4^+^ T cells following HTNV infection. Further analysis validated that the trend of CD4^+^ T cells changed in the opposite direction to that of CD8^+^ T cells in a same patient (**Supplementary Figure S2**). Correspondingly, a sharp decrease in the ratio of CD4^+^ to CD8^+^ T cells was observed during the fever and oliguria phases, even reaching baseline levels in the later stages after HTNV infection (**Supplementary Figure S3**).

Next, the repertoires of the conventional T cells were further examined by dividing them into subsets based on their expression of CCR7 and CD45RA. As shown in **Figures 1D, E**, effector memory CD8^+^ T cells (CD8^+^ Tems, CD45RA-CCR7^-^), which are essential for viral clearance, were strikingly elevated during the fever and oliguria phases following HTNV invasion. Conversely, the ratios of naïve CD8^+^ T cells (CD45RA^+^CCR7^+^) were moderately reduced during the same stages. This may be related to the fact that CD8^+^ T naïves typically differentiate into CD8^+^ Tems to eliminate infected cells upon viral infection (16). Given that CD45RA^-^CCR7^+^ central memory CD8^+^ T cells (CD8^+^ Tcms) have been shown to emerge following pathogen clearance to control systemic secondary infections (17), our data also confirmed an increase in the frequency of CD8^+^ Tcms during the polyuria and recovery stages. Terminally differentiated cells (TemRAs, CD45RA^+^CCR7^-^), a subset of T cells that re-express CD45RA, have been proved to be linked to prolonged exposure to antigens (18). In this regard, we found that the proportion of CD8^+^ TemRAs decreased in the early phases and subsequently increased in the late phases after HTNV infection (**Figures 1D, E**). For the subsets of CD4^+^ T cells, we observed a greater proportion of Tnaïves (CD45RA^+^CCR7^+^) and Tcms (CD45RA^-^CCR7^+^) throughout the course of HFRS, accompanied by a lower proportion of Tems (CD45RA^-^CCR7^-^) and TemRAs (CD45RA^+^CCR7^-^) (**Figures 1F, G**).

We then examined the activation status of CD8^+^ T cells (**Figures 1H, I**), and confirmed that CD8^+^ T cells exhibited an activated phenotype characterized by increased expression of CD38 during the fever and oliguria stages. It is noteworthy that the expression of PD-1, an inhibitory receptor for T cell exhaustion (19), also increased to suppress T cell overactivation (**Figures 1H, I**). These results suggest that CD8^+^ T cells were activated in the fever and oliguria phases after HTNV infection. Considering that regulatory T cells (Tregs) function as suppressors of T cell immunity during the acute phase of viral infection, we also analyzed the changes of Tregs, but no alteration was observed throughout the trajectory of HTNV-induced HFRS (**Supplementary Figure S4**).

### 3.2 Dynamic fluctuations of unconventional T cells in the context of HTNV infection

**Figure 2:**
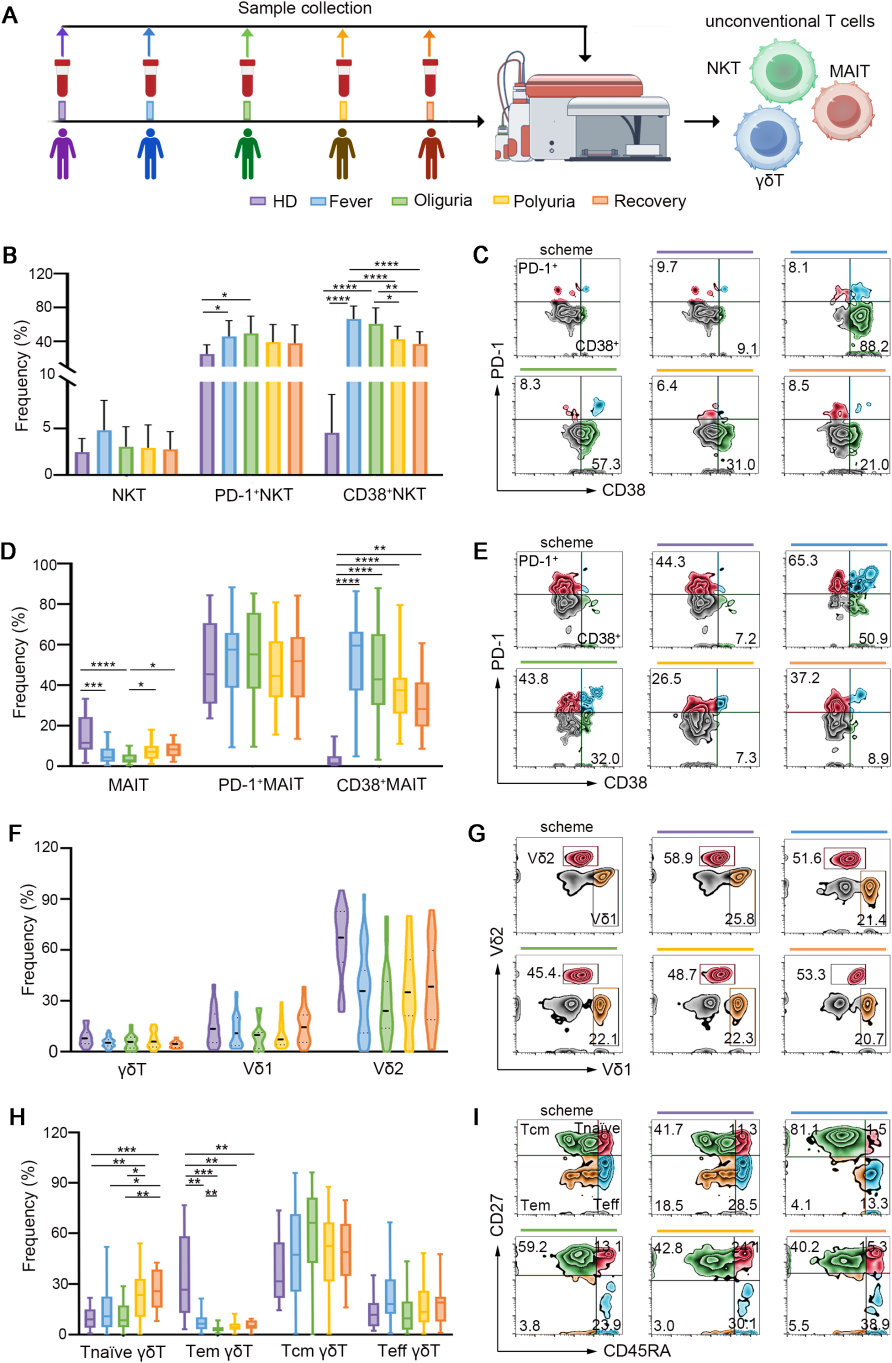
Dynamic fluctuations of unconventional T cells in HTNV-induced HFRS trajectories. **(A)** Scheme for sample collection and unconventional T cell detection. **(B)** Frequencies of total NKT cells and activated NKT cells during different clinical phases of HFRS. **(C)** Representative flow-cytometric plots of activated NKT cell subsets (gated on NKT cells). **(D)** MAIT cells and their activation status in the context of HFRS. **(E)** Representative flow cytometric plots showing activated MAIT cells. **(F)** Ratios of γδ T cells and their subsets following HTNV infection. **(G)** Representative flow plots depicting Vδ1 and Vδ2 (gated on γδ T cells). **(H, I)** Fluctuations of the subsets of γδ T cells in the context of HTNV-induced HFRS (gated on γδ T cells). Data are represented as mean ± SD. One-way ANOVA or the Kruskal–Wallis test with Dunn’s multiple comparison *post-hoc* test, *p< 0.05, **p< 0.01, ****p< 0.001, ****p< 0.0001.

In addition to conventional T cells, unconventional T cells, which are responsible for 10% of circulating T cells, have also captured considerable attention for their crucial functions in antiviral immunity (20). There are three major types of unconventional T cells including natural killer T cells (NKTs), mucosal-associated invariant T cells (MAITs) and γδ T cells. NKTs, a subset of T cells exhibiting properties of innate NK cells, were distinguished by their coexpression of CD3 and CD56. Upon stimulation, NKT cells secrete interferon-gamma (IFN-γ) and interleukin-4 (IL-4), thereby facilitating the function of T and B cells in antiviral immune responses (21). Our findings revealed that NKTs were activated during the fever and oliguria stages, evidenced by increased expression of CD38 (**Figures 2A-C**). Interestingly, the frequency of NKT cells showed a same fluctuation trend as that of CD4^+^ T cells, but exhibited an opposite change trend as that of CD8^+^ T cells in a same patient (**Supplementary Figure S2**).

MAIT cells, which are characterized by high expression of C-type lectin CD161 on CD8^+^ T cells, account for 10% of circulating human T cells (22). MAIT cells serve as the primary source of IFN-γ production in response to IL-12 and IL-18 stimulation, and their reduction has been observed in various viral infections (22–25). As shown in **Figures 2D, E**, our results revealed that MAIT cells were reduced during the fever and oliguria phases after HTNV infection, but the remaining MAIT cells were activated as evidenced by increased CD38 expression. When the disease progressed to the polyuria and recovery stages, both the frequency and activation of MAIT cells were restored. Notably, in a same patient, the ratio of MAIT cells fluctuated in a trend opposite to that of CD8^+^ T cells (**Supplementary Figure S2**). This may suggest differing temporal patterns in their functional activation. Specifically, CD8^+^ T cells typically undergo rapid expansion during acute viral clearance, whereas MAIT cells may exhibit delayed proliferation during the convalescent phase, to modulate tissue repair and immune resolution.

γδ T cells, a subset of lymphocytes expressing γδ TCRs rather than αβ TCRs, play essential roles in controlling viral infections (26, 27). Vδ1 and Vδ2 are the two major subsets of γδ T cells (28). In this study, no significant difference was found in the ratio of Vδ1 and Vδ2 after HTNV infection (**Figures 2F, G**). Interestingly, a notable decrease in γδ^+^ Tem cells and an increase in γδ^+^ T naïve cells were observed throughout the whole course of HTNV-induced HFRS (**Figures 2H, I**).

### 3.3 Dynamic changes of B and NK cells following HTNV infection

**Figure 3:**
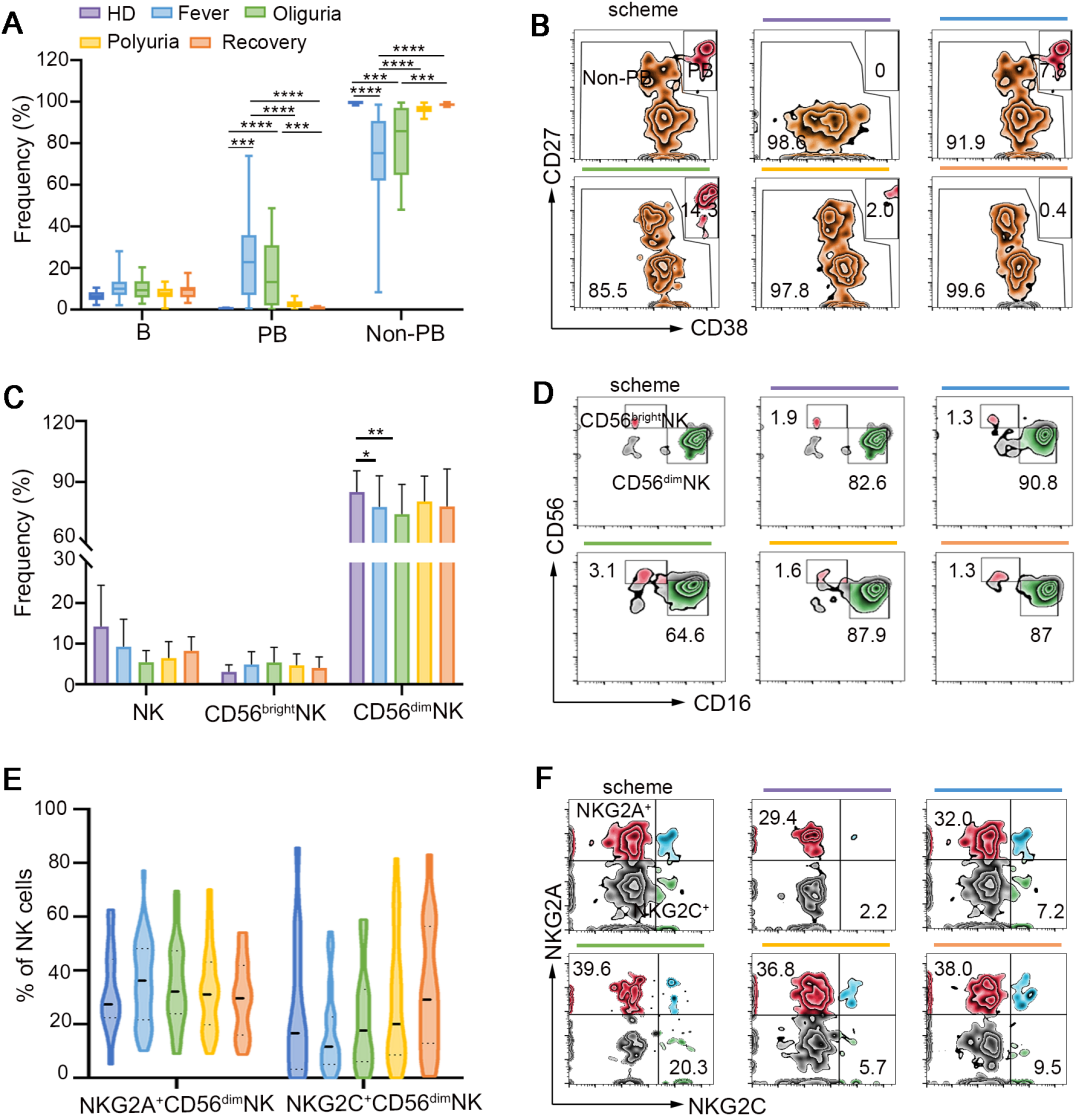
B and NK cell responses following HTNV infection. **(A)** Frequencies of B cells, PB cells and non-PB cells during different clinical phases of HTNV-induced HFRS. **(B)** Representative flow cytometric plots defining PB cells and non-PB cells (gated on B cells). **(C)** Perturbations of NK cells and their repertoires in the context of HFRS. **(D)** Representative flow-cytometric plots of CD56^dim^NK and CD56^bright^NK cells in each group. **(E, F)** The activation and exhaustion of NK cells following HTNV infection (gated on NK cells). Data are represented as mean ± SD. One-way ANOVA or the Kruskal–Wallis test with Dunn’s multiple comparison *post-hoc* test, *p< 0.05, **p< 0.01, ***p< 0.001, ****p< 0.0001.

Rapid and efficacious humoral response is one of the most important parts for virus clearance. However, few studies have examined the dynamic B cell response in the context of HTNV infection. Our findings revealed that no significant differences were discerned in B cell frequency in the context of HTNV infection. However, plasmablasts (PBs), which are differentiated from B cells and act as an integral part of a protective antiviral response (29), experienced a roughly 100-fold increase during the fever and oliguria phases, and gradually decreased to the baseline levels by the end of HTNV-induced HFRS (**Figures 3A, B**), suggesting the mobilization of PBs during the initial stage of HTNV infection.

Natural killer (NK) cells can rapidly respond to viral infections by releasing cytotoxic granules, including granzymes and perforin (9). Our findings confirmed that CD56^dim^ NK cells constituted the predominant subset of circulating NK cells (**Figure 3C**). A decline in the proportion of CD56^dim^ NK cells was observed during the fever and oliguria stages (**Figures 3C, D**). However, no significant alterations were detected in the expression of activating receptor NKG2C and the inhibitory receptor NKG2A on CD56^dim^ NK cells. Also, the frequency of CD56^bright^ NK cells was not affected during HTNV-induced HFRS (**Figures 3E, F**).

### 3.4 Phase-specific lymphocyte subsets predict disease severity and reveal immunopathogenic hallmarks in HFRS

**Figure 4:**
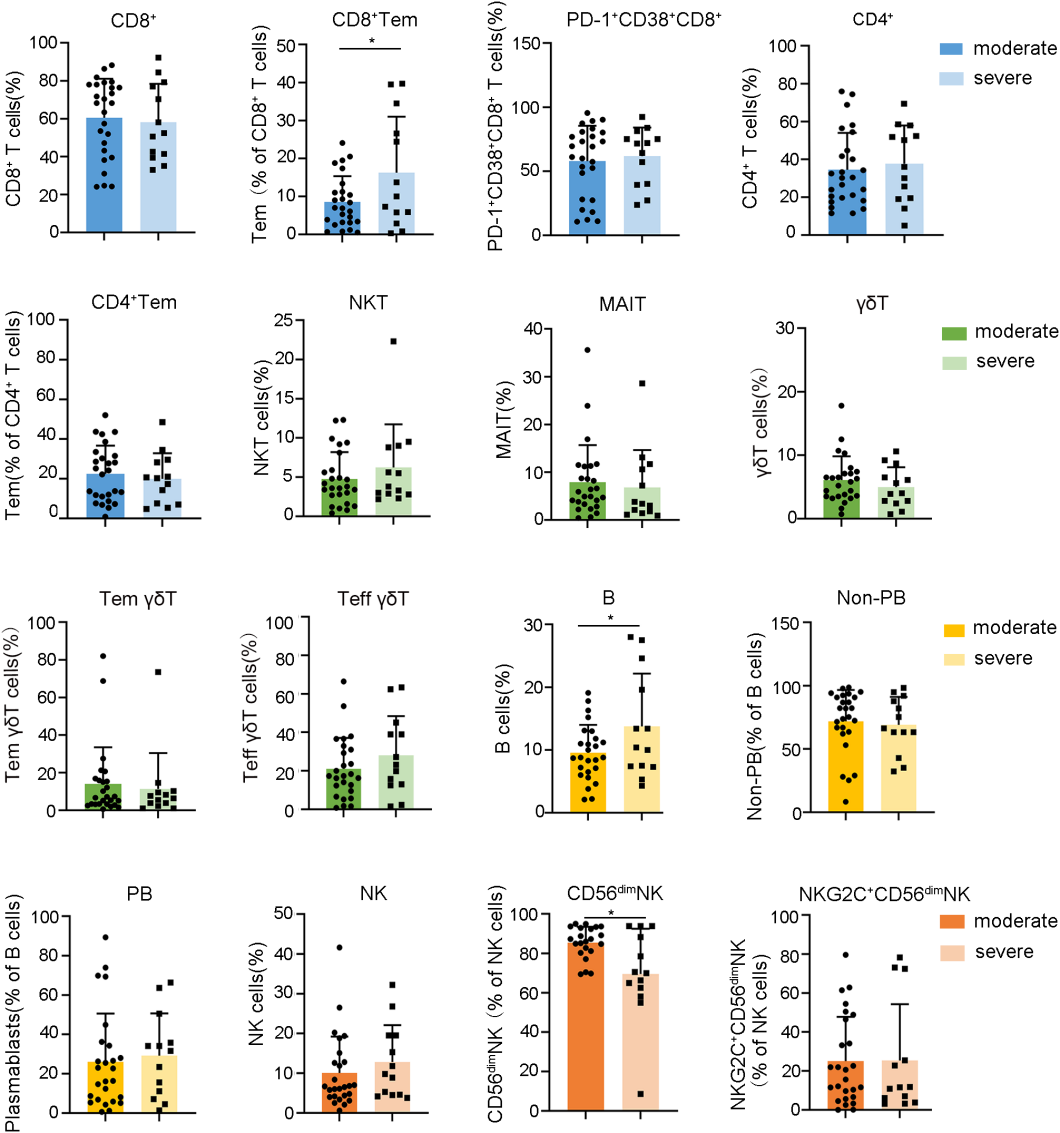
Relationships between the frequencies of lymphocyte subsets during the fever stage and HFRS severity. Data are represented as mean ± SD. Mann–Whitney U test *post-hoc* test. *p< 0.05.

After mapping the dynamic landscape of lymphocyte repertoires, we further investigated whether the frequencies of these altered subsets in the fever stage were different between moderate and severe cases of HFRS. Our data identified a notable increase in the proportions of CD8^+^ Tem and B cells in severe HFRS patients compared to those with moderate symptoms, whereas the frequencies of CD56^dim^ NK cells were found to be decreased in severe cases relative to their moderate counterparts (**Figure 4**, **Supplementary Figures S5**-**S7**).These findings enable early risk stratification to facilitate timely intervention for potentially severe cases.

**Figure 5:**
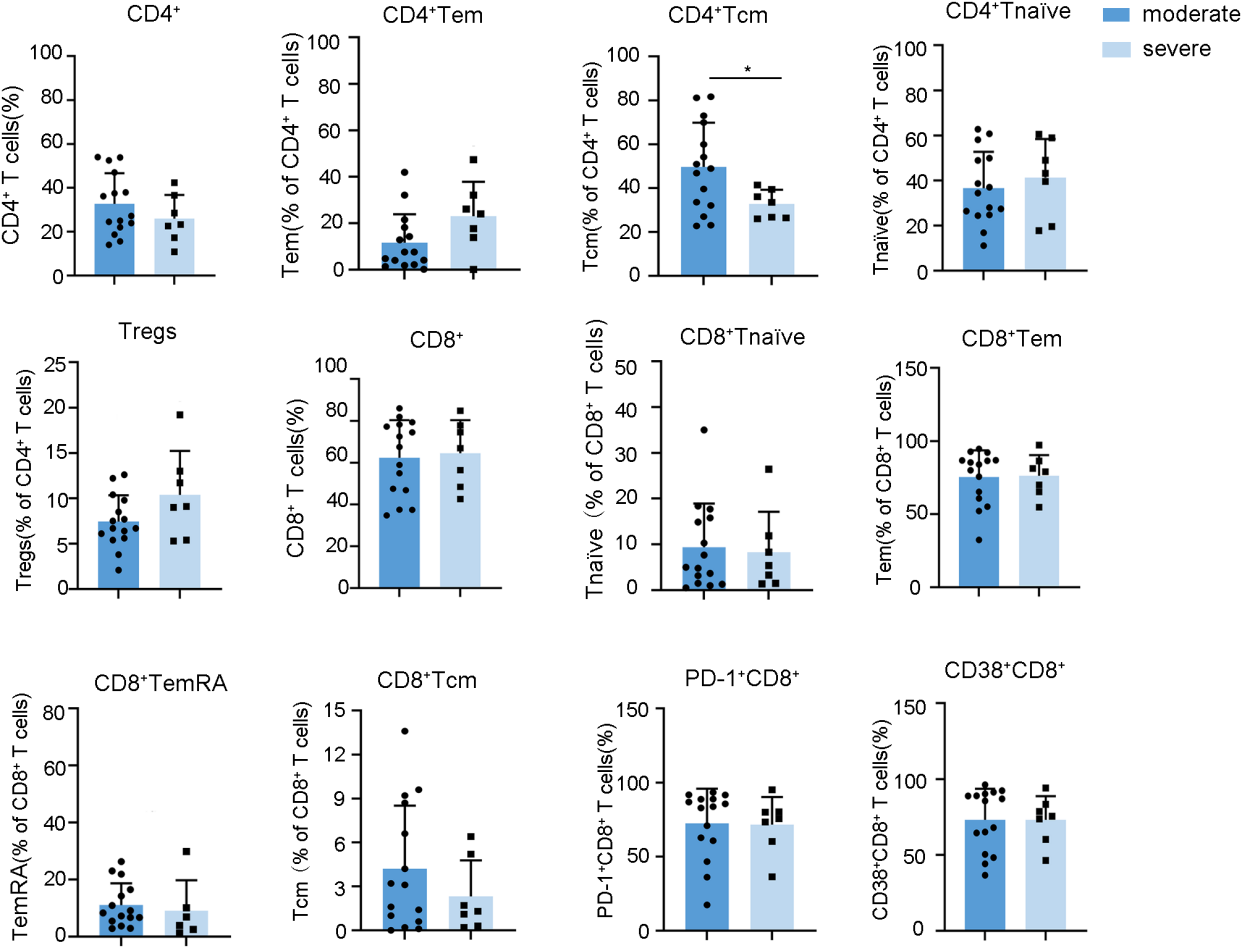
Relationships between the frequencies of lymphocyte subsets during the oliguria stage and HFRS severity. Data are represented as mean ± SD. Mann–Whitney U test *post-hoc* test. *p< 0.05.

In light of the oliguric phase, characterized by peak renal injury and immune dysregulation, being critical for understanding the immunopathogenesis of HFRS, we conducted a comparative analysis of lymphocyte profiles in patients with severe versus moderate HFRS during this phase. Our findings notably indicated that patients with severe HFRS exhibited significantly lower frequencies of CD4^+^ Tcm (**Figure 5**, **Supplementary Figures S8**, **S9**). Considering the essential function of CD4^+^ Tcm in sustaining long-term immunological memory through their self-renewal capacity and potential for rapid effector differentiation (30), the observed decrease in CD4^+^ Tcm among severe HFRS patients during the oliguric phase may suggest compromised immunological memory formation or excessive effector differentiation. This observation identifies diminished CD4^+^ Tcm levels as a crucial immunological marker for the progression of HFRS to severe disease. Moreover, although both serum Creatinine (CREA) and Urea also demonstrated strong correlations with the severity of HFRS during the oliguric phase (**Supplementary Figure S10**), Spearman’s analysis revealed no significant correlation between oliguric-phase CD4^+^ Tcm levels and creatinine or urea (**Supplementary Figure S11**). This suggests that the reduction of CD4^+^ Tcm may reflect systemic immunopathology rather than direct renal injury. These findings implied that strategies focused on enhancing CD4^+^ Tcm generation or inhibiting excessive effector differentiation could potentially facilitate improved immune reconstitution in patients with severe HFRS. We also conducted a comparative analysis of the dynamic changes in lymphocytes between severe and moderate HFRS groups. However, no significant differences were observed (**Supplementary Figures S12**-**S14**).

### 3.5 CD38 and CD161 are potential biomarkers for predicting the dynamic fluctuations of indicated lymphocyte subsets in the context of HTNV-induced HFRS

**Figure 6:**
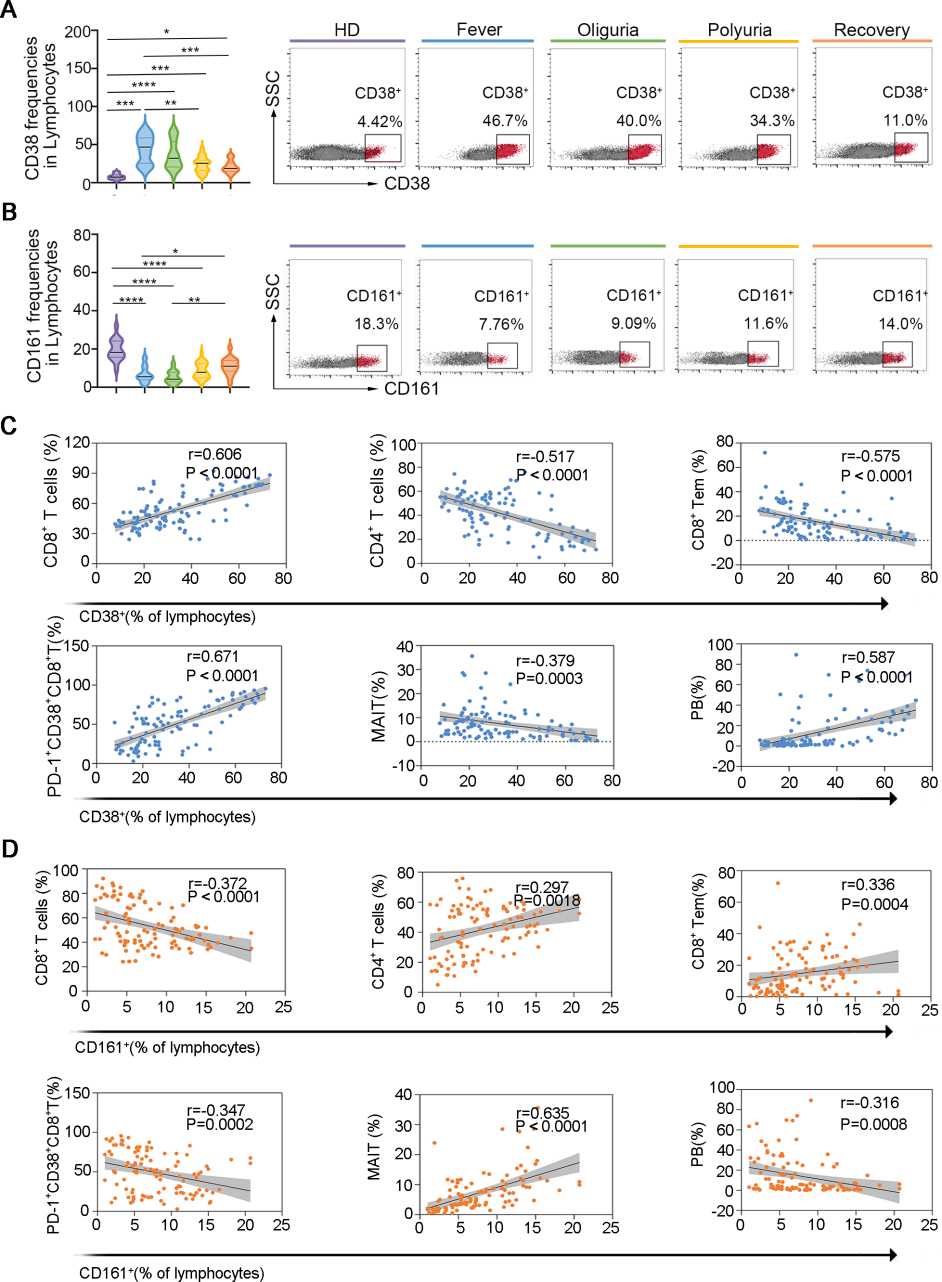
CD38 and CD161 as potential biomarkers for predicting dynamic perturbations of specific lymphocyte subsets during the progression of HTNV-induced HFRS. (A. B) The expression of CD38 **(A)** and CD161 **(B)** on circulating lymphocytes. **(C, D)** Spearman correlations of altered lymphocyte subsets and CD38 **(C)** and CD161 **(D)** expression. Data are represented as mean ± SD. One-way ANOVA or the Kruskal–Wallis test with Dunn’s multiple comparison *post-hoc* test, *p< 0.05, **p< 0.01, ***p< 0.001, ****p< 0.0001.

CD38, CD161, CD27, CD127 and CCR7 are receptors that are extensively expressed on multiple lymphocyte subsets and have been demonstrated closely related to various diseases, including viral infections, autoimmune diseases and cancers (31–35). To investigate whether the expression of these receptors is related to the progression of HTNV-induced HFRS, we analyzed the expression of these receptors on circulating lymphocytes. Our findings revealed that the expression of CD27, CD127 and CCR7 were not affected during the fever and oliguria stages of HFRS (**Supplementary Figure S15**). However, the expression of CD38, which is highly expressed on T cells and plasma cells, was found to be increased during the fever and oliguria stages, but decreased to the baseline levels during the polyuria and recovery stages (**Figure 6A**).

Conversely, the proportion of CD161, which is expressed at high levels on CD8^+^ T cells, CD4^+^ T cells, NK cells, NKT and MAIT cells, decreased notably during the fever and oliguria stages but increased during the polyuria and recovery stages (**Figure 6B**). Further analysis revealed that the frequencies of CD8^+^ T cells, CD8^+^ Tems, activated CD8^+^ T -cells, activated MAIT cells, activated NKT cells, as well as PBs were positively correlated with CD38 expression and negatively correlated with CD161 expression. Correspondingly, the ratios of CD4^+^ T and MAIT cells exhibited strong positive correlations with CD38 expression but negative correlations with CD161 (**Figures 6C, D**). The identification of CD38 and CD161 as predictors of specific lymphocyte fluctuations may inform future immunomodulatory strategies and therapeutic monitoring approaches for HFRS.

## 4 Conclusion and discussion

In this study, we depicted the dynamic landscape of circulating lymphocytes during the trajectory of HFRS caused by HTNV. Our findings revealed a notable increase in the frequencies of CD8^+^ T cells, CD8^+^ Tems, and activated CD8^+^ T, MAIT and NKT cells during the fever and oliguria stages, followed by a decrease during the polyuria and recovery stages. Conversely, the proportions of CD4^+^ T and MAIT cells exhibited opposite fluctuation patterns compared to those of CD8^+^ T cells. The frequencies of γδ^+^ Tems and CD4^+^ Tems declined during HTNV-induced HFRS. Further analysis discriminated that (1): higher frequencies of CD8^+^ Tem, B and CD56^dim^ NK cells at the fever stage were closely related to the severity of HFRS, thereby enabling early risk stratification to facilitate timely intervention for potentially severe cases; (2) lower levels of CD4^+^ Tcm cells during oliguria as a critical immunological hallmark of HFRS progression to severe disease, suggesting that strategies aimed at enhancing CD4^+^ Tcm generation or inhibiting excessive effector differentiation may improve immune reconstitution in patients with severe HFRS; (3) The identification of CD38 and CD161 as predictors of specific lymphocyte fluctuations may inform future immunomodulatory strategies and therapeutic monitoring approaches for HFRS.

Focusing on the similarities between HTNV and PUUV infection, we found HTNV-induced HFRS shows several lymphocyte immune characteristics analogous to those in PUUV-induced HFRS, such as a robust CD8^+^ T cell expansion, a potent plasmablast response, and a marked decrease and activation of MAIT cells at the onset of viral infection (10, 36). This phenomenon may be attributed to the high similarities between HTNV and PUUV. Genomically, HTNV and PUUV are all negative-sense, single-stranded RNA viruses composed of three gene segments: small (S), medium (M), and large (L), which encode the nucleocapsid protein, the Gn and Gc glycoproteins, and the RNA-dependent RNA polymerase, respectively (37–39). Morphologically, HTNV and PUUV are predominantly spherical, with diameters ranging from 80 to 150 nm (40, 41). It is worth noting that the tetrameric Gn-Gc spike complex structure has been identified in both HTNV and PUUV (40–42), and the crystal structures of the Gn glycoprotein are remarkably similar (43, 44).

It is also important to highlight the differences in the lymphocyte responses elicited by HTNV and PUUV infections. Firstly, although Tregs have been shown to be elevated in PUUV infection (45), our study did not corroborate this finding in patients with HTNV-induced HFRS (**Supplementary Figure S4**). Secondly, prior research indicates that NK cells undergo expansion and maintain elevated levels for more than 60 days after PUUV infection (46). However, no significant difference in NK cell frequency was observed in the context of HTNV infection (47). Thirdly, in contrast to earlier finding that reported the activation of CD56^dim^ NK during the acute phase of PUUV-induced HFRS (47), our investigation revealed that the expression levels of the activating receptor NKG2C and the inhibitory receptor NKG2A on CD56^dim^ NK cells remained unaffected in HTNV-induced HFRS (**Figures 3E, F**). Furthermore, while previous studies have demonstrated the correlation between CD8^+^ T cells and activated MAIT cells with HFRS severity (36, 48, 49), such associations were not confirmed in the present study (**Figure 4**). The distinct characteristics of the two viruses may account for these discrepancies. Epidemiologically, HTNV is a murine-borne hantavirus prevalent in Asia, whereas PUUV is an arvicoline-borne hantavirus mainly reported in Europe (50, 51). Structurally, the Gc protein, which is integral to receptor binding and membrane fusion and is also regarded as the primary source of neutralizing antibody production, exhibits a low level of conservation between HTNV and PUUV (41). Notably, HFRS caused by HTNV is more severe than that caused by PUUV infection, which typically presents with a milder clinical course (1, 2). The combined effects of these factors may contribute to the differences in lymphocyte landscapes between HTNV and PUUV-induced HFRS. In this regard, in the follow-up, more research efforts should be devoted to elucidating the underlying mechanisms responsible for the distinct immunophenotypes induced by different hantaviruses. Furthermore, considering the key role of cytokines secreted by lymphocytes in the antiviral response, a comprehensive investigation of the effect of alterations in lymphocyte subsets on cytokine secretion is also very necessary.

CD38 is involved in multiple inflammatory processes, encompassing migration, aggregation, adhesion, phagocytosis, and antigen presentation. An increase in CD38^+^ immune cells has been documented in various viral infections, such as HIV, COVID-19, and Epstein-Barr virus (EBV) infections (52–54). Upon activation and migration, both T and B cells exhibit increased CD38 expression, whereas NK cells sustain a constant level of CD38 expression, consistent with our research findings. Additionally, CD38 may also impact disease severity, potentially playing a pivotal role in exacerbating symptoms of SARS-CoV-2 infection and elevating the risk of secondary bacterial infections (55, 56). Furthermore, studies related to HIV indicate that CD38 indirectly reflects viral load, rendering cells more susceptible to HIV infection and promoting HIV replication (57). These findings suggest the potential utility of CD38 as an effective biomarker in viral infectious diseases. Another noticeable fact is that CD38 is expressed not only on lymphocytes but also on dendritic cells, neutrophils and monocyte macrophages under inflammatory conditions (58). Given that CD38 can predict the dynamic fluctuations of indicated lymphocyte subsets, our subsequent research will focus on examining the immunophenotype of dendritic cells, neutrophils and monocyte macrophages in HTNV-induced HFRS.

CD161 is typically expressed on monocytes, NK cells, NKT cells, and a subset of peripheral blood T lymphocytes, with its high expression levels being mainly composed of a population of MAIT cells (59). Several reports have demonstrated that CD161 functions as an inhibitory receptor on NK cells, where it binds to specific ligands to attenuate their cytotoxic activity. During viral infections, CD161 serves as a marker of pro-inflammatory NK cell function. For instance, the downregulation of CD161 expression has been correlated with increased liver inflammation in chronic hepatitis C virus (HCV) infection. Furthermore, the lack of CD161^+^ NK cell subsets in HIV-infected individuals may contribute to decreased proliferative capacity in response to homeostatic cytokines and impaired ability to secrete IFN-γ (60).The extent of CD161 effect on T cells remains elusive, with reports suggesting both costimulatory and inhibitory effects (61).

Collectively, our findings shed light on the trajectory of circulating lymphocytes during HTNV infection, discriminate the lymphocyte subsets closely related to disease severity, and identify potential biomarkers for predicting the dynamic fluctuations of indicated lymphocyte subsets, thereby providing valuable insights into the pathogenesis of HFRS.

Despite these advances, several limitations should be acknowledged in this study. First, the sample size was constrained by the seasonal epidemiology of HFRS in Shaanxi Province (peak incidence in November–February), heterogeneous clinical presentations (e.g., patients with severe HFRS usually exhibit overlapping clinical courses, while those with moderate forms of the disease often do not experience oliguria.), and practical challenges (e.g., premature patient transfers). Second, blood samples collected according to clinical phase transitions rather than at predetermined time intervals may not capture intra-phase variations, future studies could benefit from integrating phase-triggered sampling with monitoring at three-day intervals to enhance data granularity. Third, analyses of rare unconventional T cell subsets (e.g., NKT, MAIT) and their subpopulations require validation in expanded cohorts with larger blood volumes due to limited statistical power. Fourth, while we employed surface markers to identify unconventional T cells (e.g., NKT, MAIT) in this study, future work should incorporate gold-standard tetramer validations (e.g., MR1/MAIT, CD1d/NKT) to confirm their antigen-specific identities. Fifth, the lack of HTNV viral load data limits mechanistic insights, and subsequent studies should integrate viral, immune, and clinical factors to clarify the determinants of disease severity. Finally, our failure to replicate previously reported findings, such as the associations between CD8^+^ T/MAIT cells and disease severity, may be partially attributed to the limitations in cohort size rather than a lack of biological relevance, emphasizing the need for targeted validation in larger populations.

## Data Availability

The datasets presented in this article are not readily available because the corresponding authors can provide the data supporting the findings of this study upon reasonable request. Requests to access the datasets should be directed to lixiaojiao@xjtu.edu.cn.
