# Supplementary material for: Dynamic alterations of circulating lymphocytes during the trajectory of Hantaan virus-induced hemorrhagic fever with renal syndrome
Source: Front Immunol. 2025 May 29;16:1567306. doi: 10.3389/fimmu.2025.1567306 (PMC12159035; doi:10.3389/fimmu.2025.1567306)
Supplement: Supplementary file 1 [file DataSheet1.pdf]

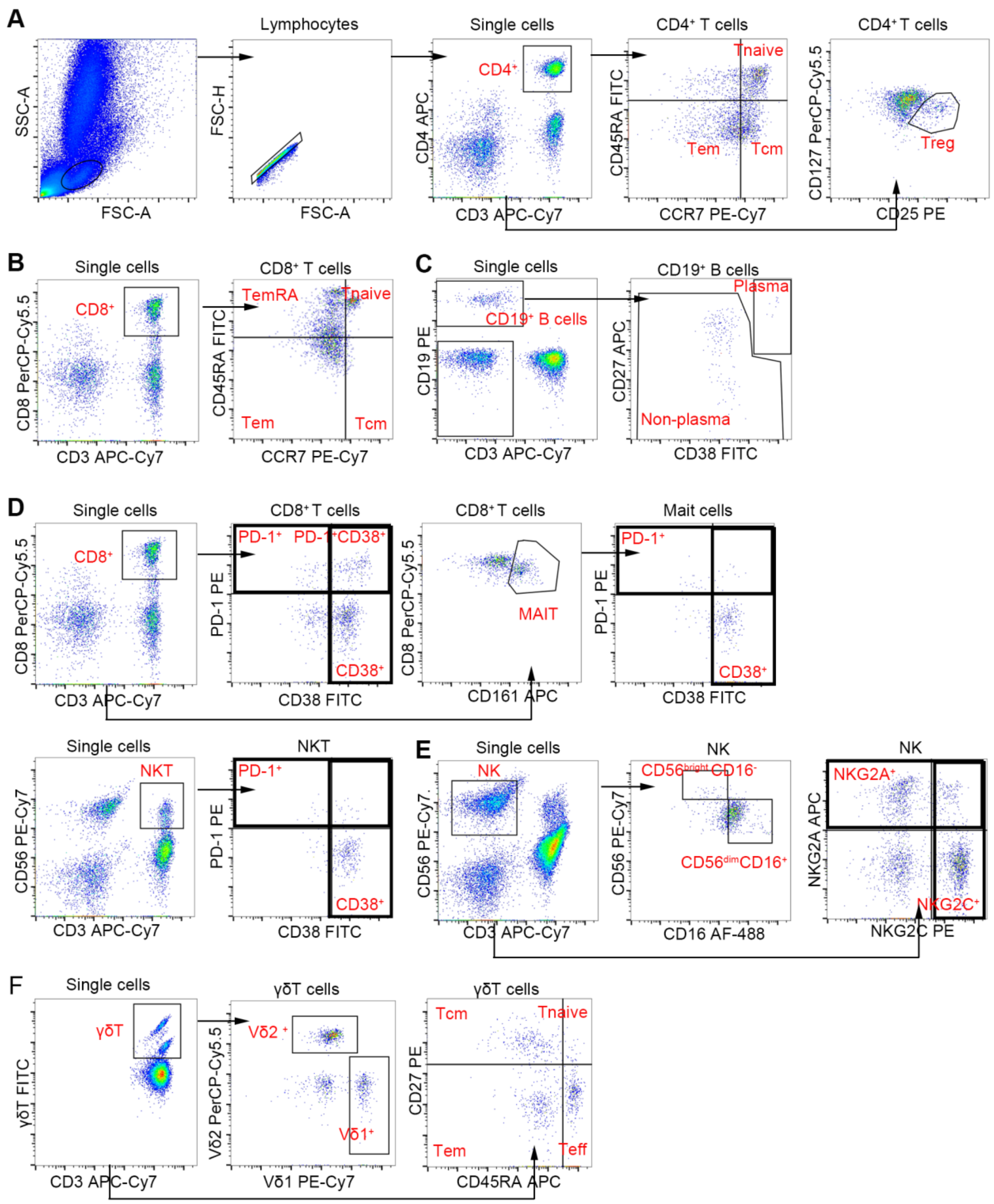

Figure S1 Flow cytometric strategy for definition of specific lymphocyte subsets.

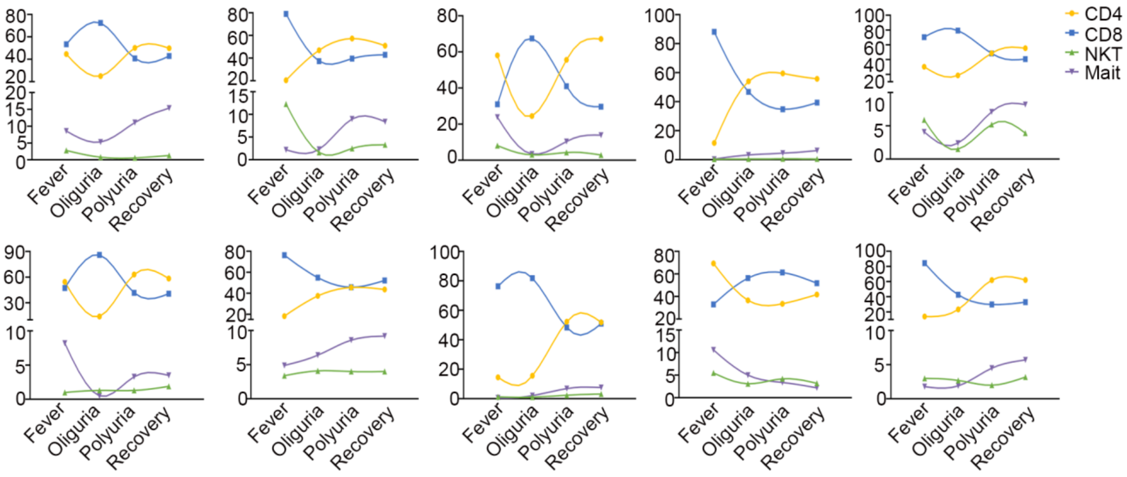

Figure S2 The dynamic trajectories of CD4$^+$ T cells, CD4$^+$ T cells,NKT cells and Mait cells in each individual patient.

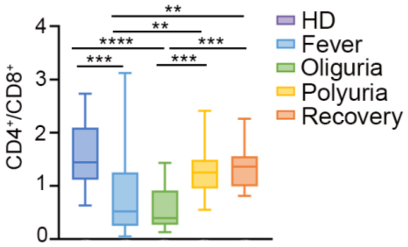

Figure S3 The ratios of CD4$^+$/CD8$^+$ T cells in the progression of HTNV-induced HFRS

Data are represented as mean ± SD. One-way ANOVA or the Kruskal–Wallis test with Dunn's multiple comparison post-hoc test, **p< 0.01, ***p< 0.001, ****p< 0.0001.

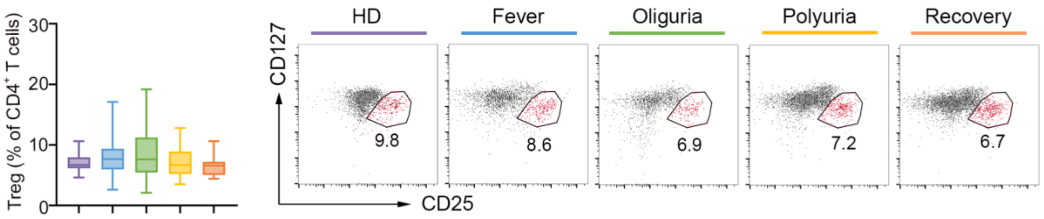

Figure S4 The proportion of Tregs in the context of HTNV-induced HFRS

(A) The frequencies of Tregs in each group. (B) The representative FACS analysis of Treg phenotype (gated on $CD4^+$ T cells). Data are represented as mean ± SD. The Kruskal–Wallis test with Dunn's multiple comparison post-hoc test, nonsignificant difference.

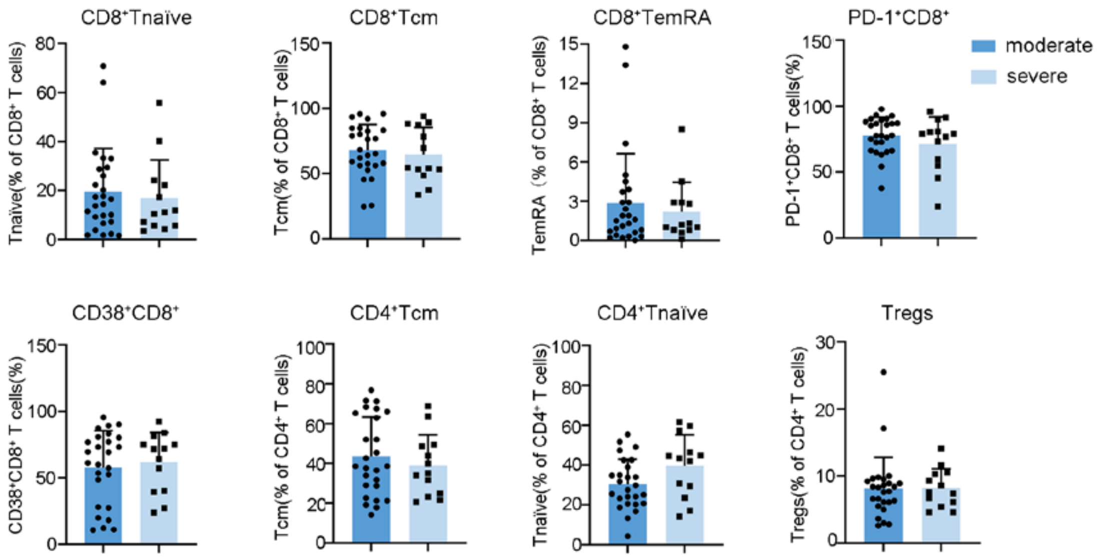

Figure S5 Relationships between the frequencies of conventional T cell subsets during the fever stage and HFRS severity

No differences were significant by the Mann–Whitney U test.

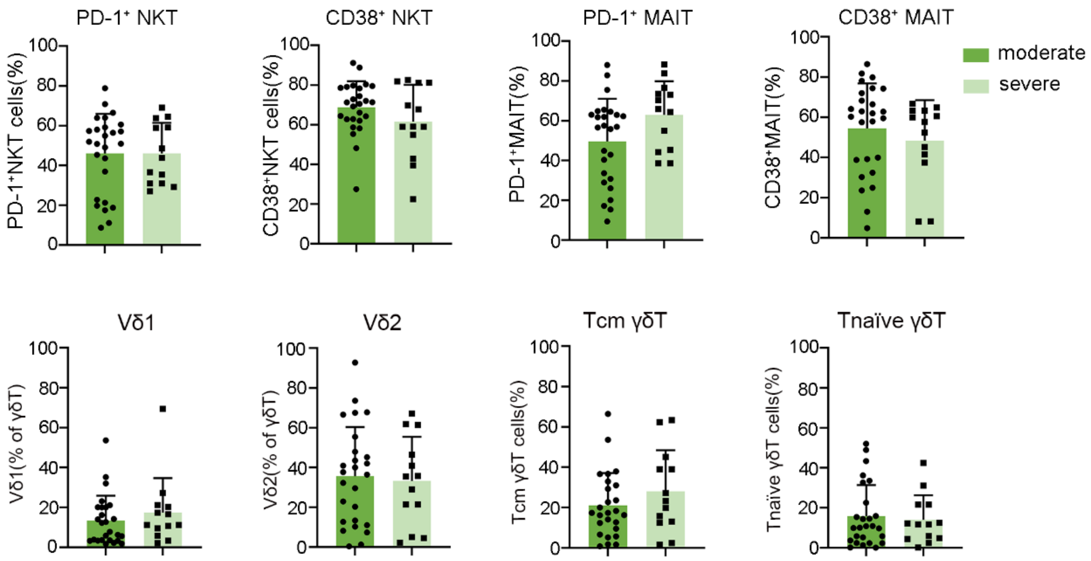

Figure S6 Relationships between the frequencies of unconventional T cell subsets during the fever stage and HFRS severity

Data are represented as mean ± SD. No groups were significantly different by the Mann–Whitney U test.

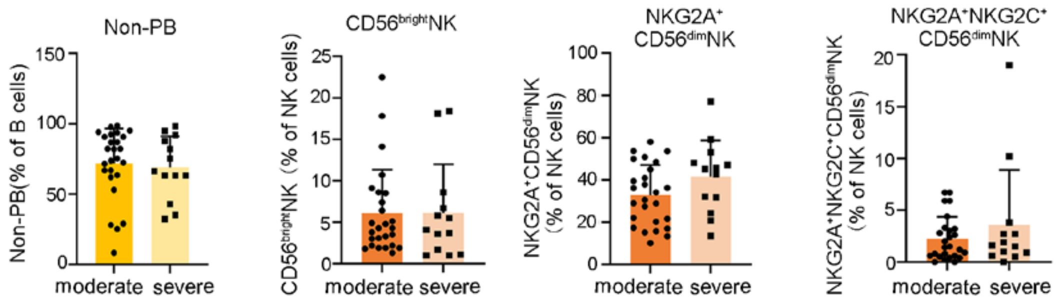

Figure S7 Relationships between the frequencies of B and NK cell subsets during the fever stage and HFRS severity

Data are represented as mean ± SD. Mann–Whitney U test post-hoc test. No statistical significance between groups was observed.

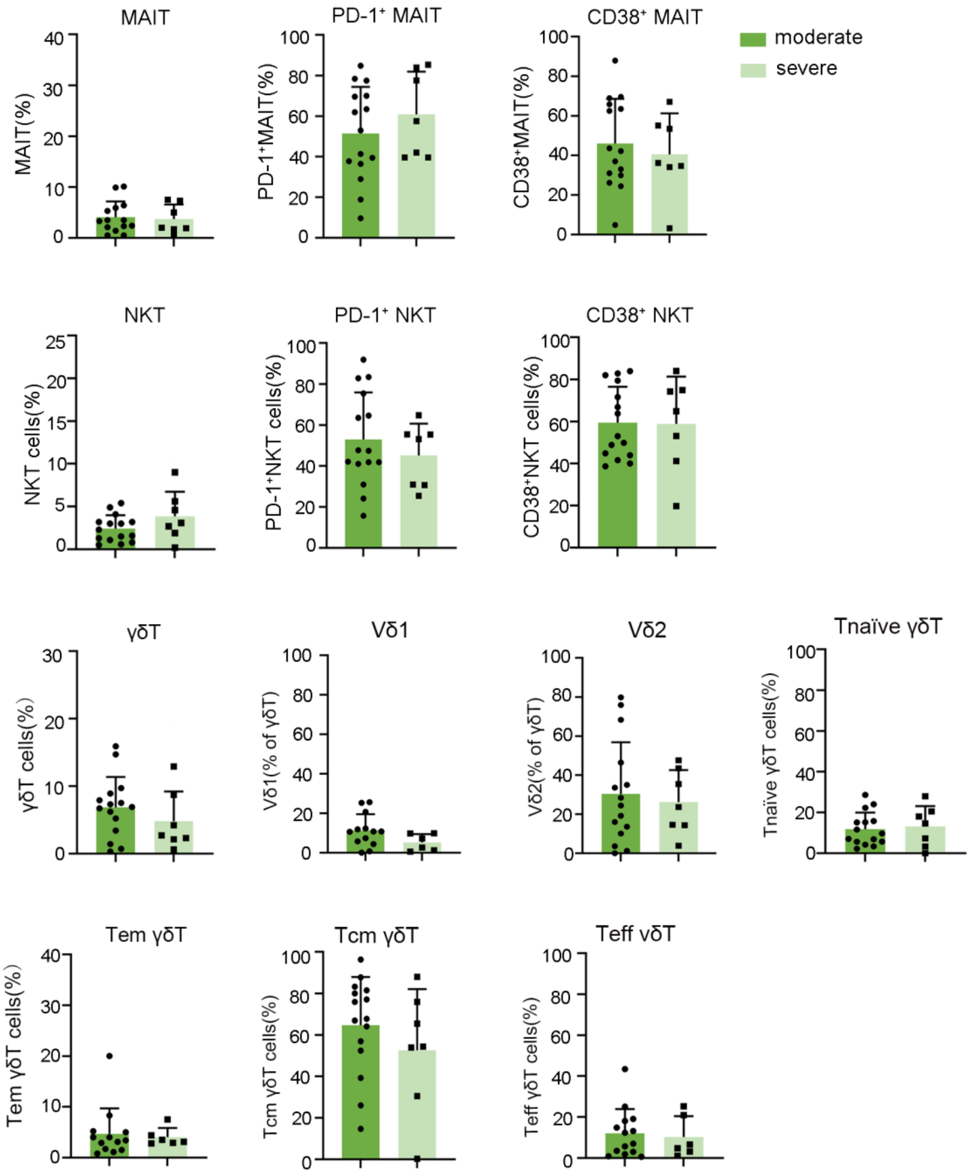

Figure S8 Relationships between the frequencies of unconventional T cell subsets during the oliguria stage and HFRS severity

Data are represented as mean ± SD. No groups were significantly different by the Mann–Whitney U test.

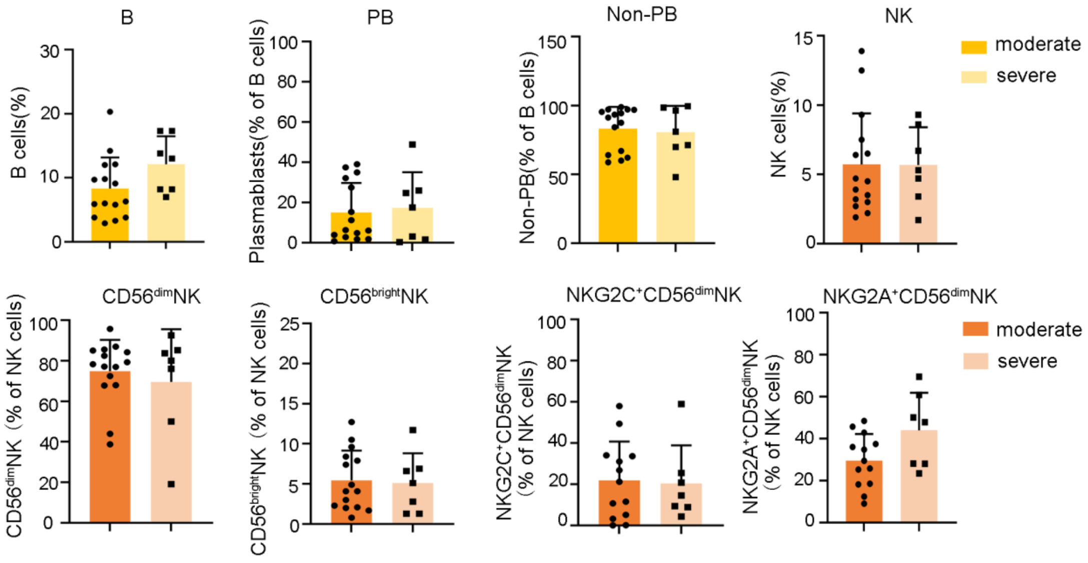

Figure S9 Relationships between the frequencies of B and NK cell subsets during the oliguria stage and HFRS severity

Data are represented as mean ± SD. No groups were significantly different by the Mann–Whitney U test.

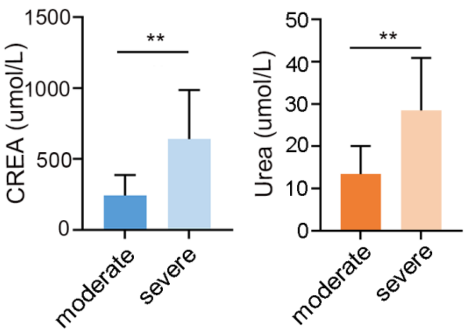

Figure S10 The relationship between the HFRS severity and creatinine or urea levels.

Data are represented as mean ± SD. Mann–Whitney U test post-hoc test. *$p < 0.01$

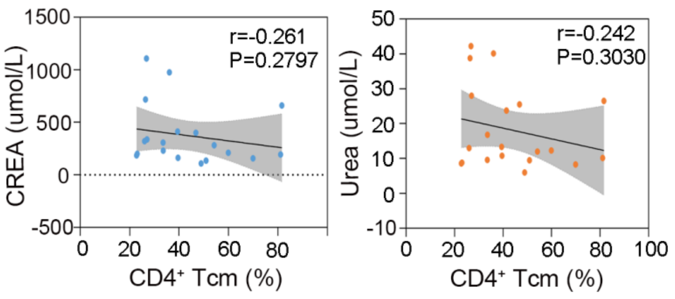

Figure S11 Spearman correlations of CD4^{+} Tcm frequencies with creatinine and urea levels during the oliguria stage.

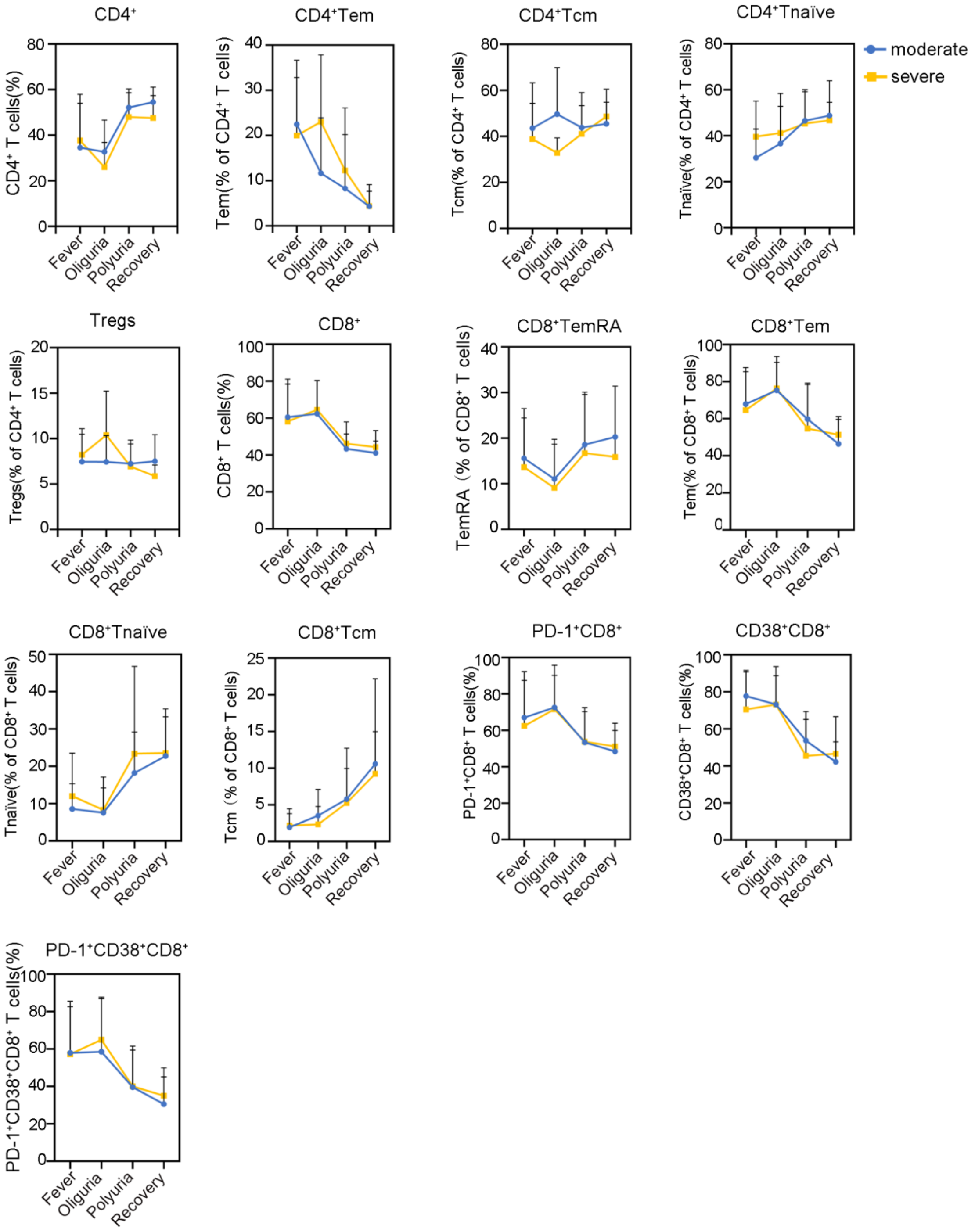

Figure S12 Contrasting temporal dynamics of conventional T cell subsets in severe and moderate HFRS.

Data are represented as mean ± SD. No groups were significantly different by the Linear mixed-effects model analysis.

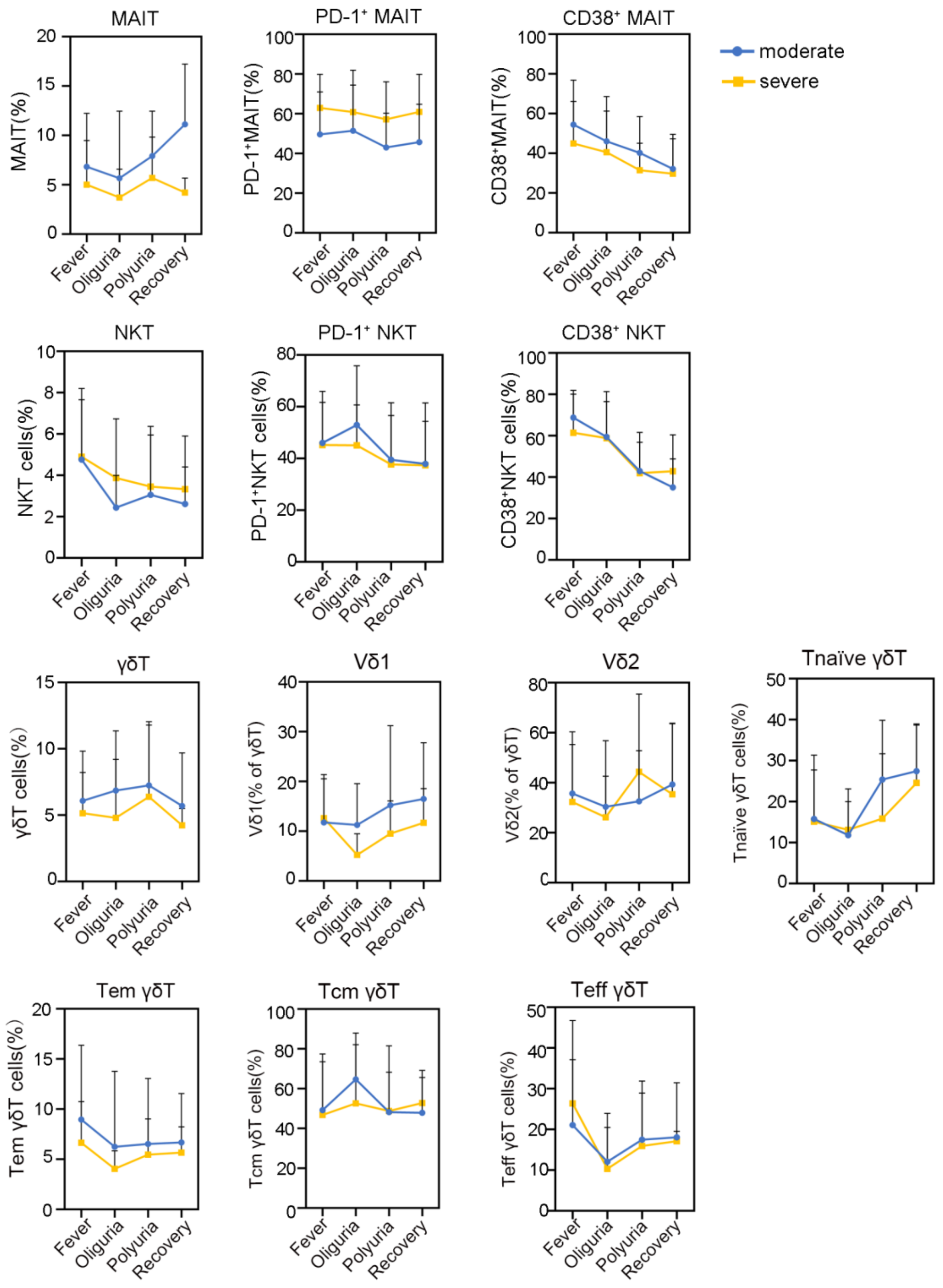

Figure S13 Contrasting temporal dynamics of unconventional T cell subsets in severe and moderate HFRS

Data are represented as mean ± SD. No groups were significantly different by the Linear mixed-effects model analysis.

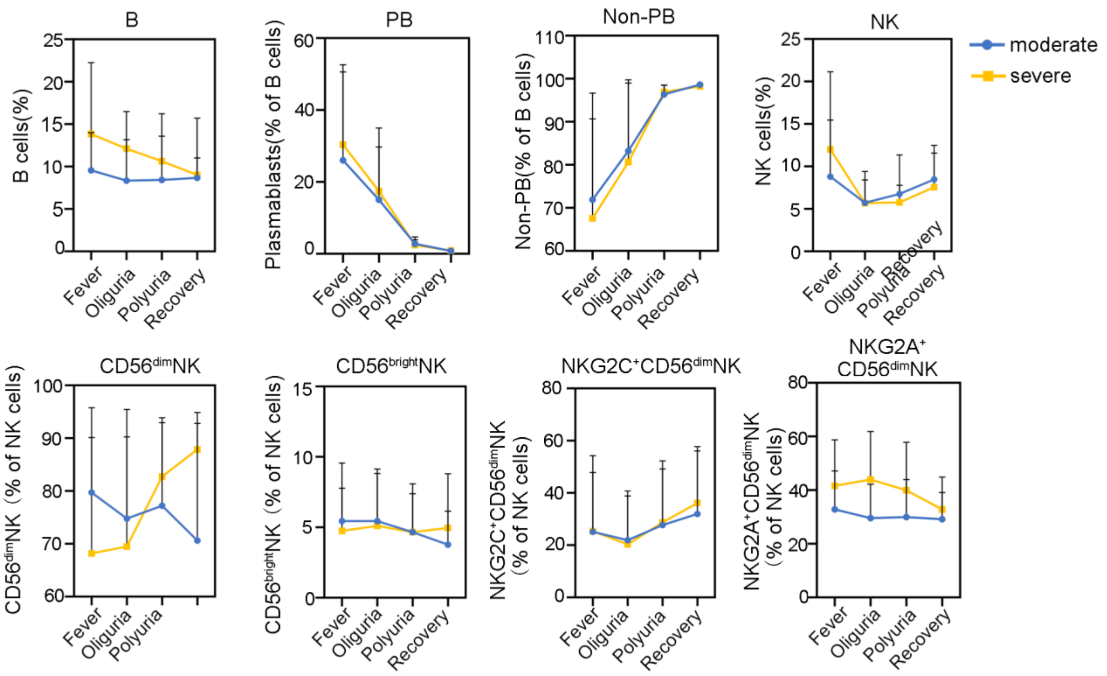

Figure S14 Contrasting temporal dynamics of B and NK cell subsets in severe and moderate HFRS

Data are represented as mean ± SD. No groups were significantly different by the Linear mixed-effects model analysis.

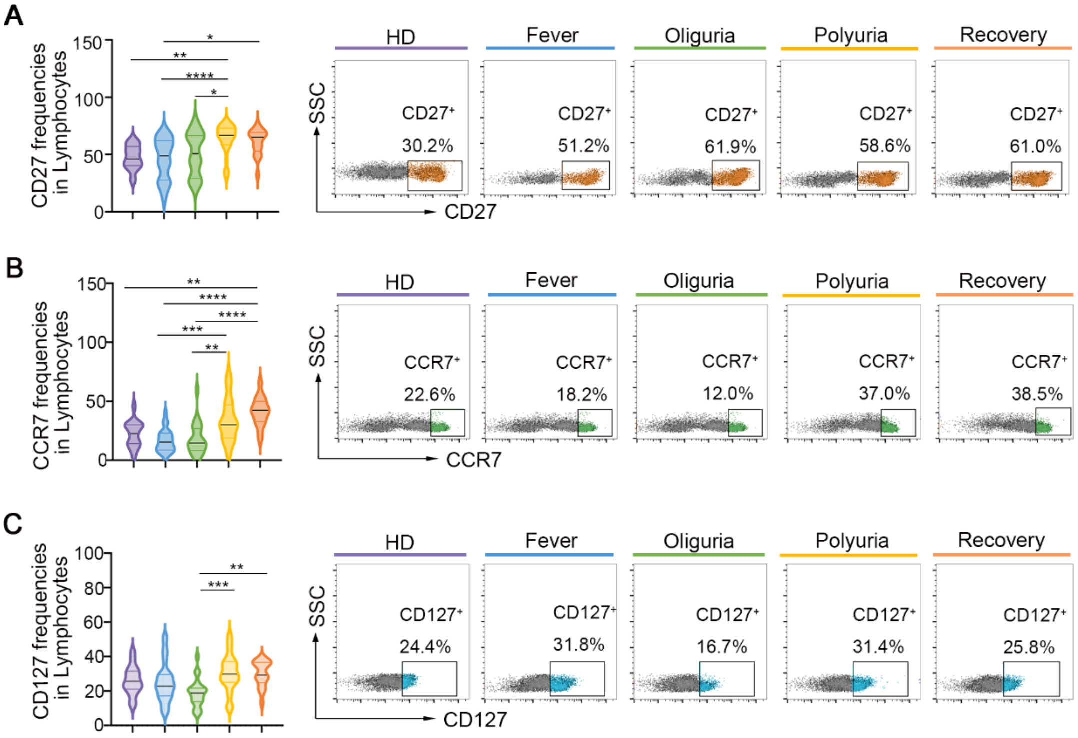

Figure S15 CD27, CCR7 and CD127 responses following HTNV infection

Data are represented as mean ± SD. One-way ANOVA or the Kruskal–Wallis test with Dunn's multiple comparison post-hoc test, *$p< 0.05$, **$p<$ 0.01, ***$p<$ 0.001, ****$p<$ 0.0001.
